# Colon Tissues Classification and Localization in Whole Slide Images Using Deep Learning

**DOI:** 10.3390/diagnostics11081398

**Published:** 2021-08-02

**Authors:** Pushpanjali Gupta, Yenlin Huang, Prasan Kumar Sahoo, Jeng-Fu You, Sum-Fu Chiang, Djeane Debora Onthoni, Yih-Jong Chern, Kuo-Yu Chao, Jy-Ming Chiang, Chien-Yuh Yeh, Wen-Sy Tsai

**Affiliations:** 1 Department of Computer Science and Information Engineering, Chang Gung University, Guishan 33302, Taiwan; d0521006@cgu.edu.tw (P.G.); d0421008@cgu.edu.tw (D.D.O.); 2 College of Medicine, Chang Gung University, Guishan 33302, Taiwan; louisyhuang@gmail.com (Y.H.); wensyt@gmail.com (W.-S.T.); 3 Department of Anatomic Pathology, Chang Gung Memorial Hospital, Linkou 33305, Taiwan; 4 Department of Neurology, Chang Gung Memorial Hospital, Linkou 33305, Taiwan; 5 Division of Colon and Rectal Surgery, Chang Gung Memorial Hospital, Linkou 33305, Taiwan; sumfuchiang@gmail.com (S.-F.C.); ufo789.ufo789@gmail.com (Y.-J.C.); jmjiang1234@yahoo.com.tw (J.-M.C.); chnyuh@gmail.com (C.-Y.Y.); 6 Graduate Institute of Clinical Medical Sciences, College of Medicine, Chang Gung University, Guishan 33302, Taiwan; 7 School of Traditional Chinese Medicine, Chang Gung University, Guishan 33302, Taiwan; 8 College of Nursing, Chang Gung University of Science and Technology, Guishan 33302, Taiwan; kuoyu915@mail.cgust.edu.tw

**Keywords:** convolutional neural networks, colon cancer, primary tumor, deep learning, transfer learning, classification, localization

## Abstract

Colorectal cancer is one of the leading causes of cancer-related death worldwide. The early diagnosis of colon cancer not only reduces mortality but also reduces the burden related to the treatment strategies such as chemotherapy and/or radiotherapy. However, when the microscopic examination of the suspected colon tissue sample is carried out, it becomes a tedious and time-consuming job for the pathologists to find the abnormality in the tissue. In addition, there may be interobserver variability that might lead to conflict in the final diagnosis. As a result, there is a crucial need of developing an intelligent automated method that can learn from the patterns themselves and assist the pathologist in making a faster, accurate, and consistent decision for determining the normal and abnormal region in the colorectal tissues. Moreover, the intelligent method should be able to localize the abnormal region in the whole slide image (WSI), which will make it easier for the pathologists to focus on only the region of interest making the task of tissue examination faster and lesser time-consuming. As a result, artificial intelligence (AI)-based classification and localization models are proposed for determining and localizing the abnormal regions in WSI. The proposed models achieved F-score of 0.97, area under curve (AUC) 0.97 with pretrained Inception-v3 model, and F-score of 0.99 and AUC 0.99 with customized Inception-ResNet-v2 Type 5 (IR-v2 Type 5) model.

## 1. Introduction

Colorectal cancer (CRC) is one of the leading causes of death worldwide. Among the incidence of cancer, Asia accounted for 49.3% of the cancer cases; moreover, more than half of the total mortality caused by cancer is 58.3%. In terms of the type of cancer, colorectal cancer accounted for 10% of newly diagnosed cases, remaining as the third leading cause of cancer death in Asia [1]. Cancer remained the top cause of death in Taiwan for the 38th year, accounting for 28.6% of the country’s total mortality in 2019. Out of the total 50,232 deaths caused by cancer, colon cancer resulted in 13% of the total cancer-related deaths [2]. The mortality may be caused due to the late diagnosis of the CRC, which results in metastasis and poor prognosis. As a result, the early diagnosis of CRC involving an endoscopic biopsy that facilitates a complete assessment of the colon tissues pattern and distribution of abnormalities plays an important role in making a definite diagnosis of CRC.

Conventionally, the pathologist examines the stained specimen on the glass slide under the microscope in case of histopathological image diagnosis. The assessment procedure for biopsy requires microscopic study, which is not only tedious but also time-consuming and expensive. Moreover, the morphological features of normal and precancerous or benign tumors are similar. Moreover, the inter-observer variability among the pathologists might be observed where quantitative estimation of the abnormal region is manual and subjective [3]. With the advancement of technology in medical science, the use of the microscope is gradually replaced by digitalization. In recent years, the entire glass slide containing the specimen is scanned and stored in the form of a digital image: named whole slide image (WSI). Using WSI, the challenges such as time-consuming and expensive microscopic studies can be overcome by automatic classification and localization of abnormalities. With the help of localized abnormalities in WSI, the assessment procedure can be made easier, faster, and lesser expensive.

Considering the WSI analysis, many recent works have proposed the use of machine learning (ML) techniques for cancer prediction and prognosis [4]. Unlike statistics, machine learning allows decision-making or inference-drawing by learning the patterns from the training examples. In particular, when considering histopathological image analysis, several ML methods are surveyed for different types of cancers such as breast cancer, colorectal cancer, and prostate cancer [5]. The authors discussed the potential usage of each work such as mitosis detection in breast cancer, gland segmentation in prostate and CRC, etc. However, the authors also stated the difficulties faced during extraction of local features, such as gray-level co-occurrence matrix (GLCM) and local binary pattern (LBP), when dealing with a very large image.

Therefore, several DL-based popular architectures were overviewed in [6] to provide insight into the use of deep learning (DL) methods for automatic feature extraction from the images. The authors in [7] surveyed different cancers such as mitosis detection, lesion recognition in breast cancer, and tumor grade classification in CRC. When focusing on DL-based histopathological image analysis [8], researchers discussed classification models for lung cancer, brain tumor, prostate cancer, etc., wherein hematoxylin and eosin (H&E) staining was used in WSI for different classification applications such as classifying cell, grading glioma, and predicting Gleason score [9].

There are many challenges encountered in real-world applications of histopathological data analysis using DL, such as high cost of image data collection, scanning of tissue slides, variation in appearances of different subjects, staining amount and imaging procedures, time-consuming, and expensive labeling of a WSI (size > 1 gigabyte). When employing DL models for medical image analysis, the use of transfer learning (TL) has become popular, wherein the pretrained architectures trained with natural image datasets such as ImageNet [10] are considered along with the corresponding frozen weights. These adopted state-of-the-art models are simply fine-tuned [11] for medical images. For instance, TL was used in a recent study for the breast cancer histology image classification considering seven variants of EfficientNets. Among the seven variants, the EfficientNet-B2 achieved an accuracy of 98.33% and a sensitivity of 98.44%. The results indicated that transferring generic features obtained from natural images to medical images through TL achieved satisfactory results in the case of EfficientNets [12]. However, the features learned during the training with the natural images are unassociated in terms of size, characteristics, and textural features, when compared with concerning medical images.

On the other hand, the work in [13] experimentally proved that TL considering a model with the same domain of the targeted image achieved better accuracy than TL considering a model with the different domain of the target dataset. When the diabetic foot ulcer dataset was considered, the proposed model achieved an F-score of 89.4% with TL from the different domain of the target dataset, and an F-score of 97.6% TL from the same domain of the target dataset. A similar investigation was carried out in [14], but considering the breast cancer dataset. The authors proposed a hybrid model of parallel convolutional layers and residual links, which was trained with the same domain transfer learning. The proposed model achieved classification accuracies of 97.4% and 96.1% on the validation set and the testing set, respectively. Addressing the image annotation issue, a novel TL approach was proposed in [15], wherein the need for data labeling was reduced by training the DL model with a large number of unlabeled images of a specific task followed by using a small number of labeled images for fine-tuning the model. The proposed model achieved F-scores of 98.53% and 97.51% for skin cancer and breast cancer, respectively. Nonetheless, on the basis of the above-discussed literature survey, it remains unclear whether the use of TL will provide better performance if the source domain is completely different from the target domain or if the source domain is same as the target domain. Moreover, it is also unclear as to whether the performance of the model derived by employing the same domain TL provides better performance over the model that is trained from the scratch [12,13,14,15].

Consequently, in this paper, DL methods were employed for the patch level classification of normal and abnormal CRC tissues in WSI. The aim of this work included multiphase analyses for colorectal tissue classification and localization. In phase one, pretrained convolutional neural network (CNN) architectures trained with different source domain were compared. In the second phase, different customized models were designed using Keras [16], following similar structures of popular models such as Visual Geometry Group (VGG) [17], Residual Networks (ResNet) [18], Inception [19,20], and Inception-ResNet-v2 [21]. The designed customized models were trained from the scratch considering the target dataset (own CRC dataset) instead of using the pretrained weights of ImageNet, and the performances of the different models were compared. In phase three, checking of the scope for further improvement of best performing customized model was conducted, improving the performance of the customized IR-v2. Ultimately, the abnormal regions were localized in the WSI in the final phase.

In this paper, we aimed to perform the classification and localization of the colorectal tissue, considering both the same domain and different domain TL, and training the models from scratch. Therefore, the contributions made in this work can be summarized as follows:

- A new dataset consisting of 297 WSI for the colon was collected and manually annotated by a well-experienced pathologist.
- Transfer learning was investigated considering the training of different CNN architectures using weights obtained from different domain datasets, and performances were recorded after hyperparameter tuning.
- Different customized CNNs models were built and trained from scratch using target dataset, and performances were recorded and investigated after hyperparameter tuning.
- Among the customized models, the top-performing model was studied further to check if the model can be further tuned to obtain the best customized model.
- The best-customized model IR-v2 Type 5 achieved an F-score of 0.99 and AUC 0.99.
- The patches classified as abnormal were localized in the WSI, which could be beneficial for pathologists to examine less area compared to the whole slide.
- On the basis of our study, we empirically proved that the customized IR-v2 Type 5 model provides better results for the CRC dataset if trained from scratch.
- The IR-v2 Type 5 model developed through this study may be deployed in different hospitals for automatic classification and localization of abnormal tissues in WSI, which can assist pathologists in making accurate decisions in a faster mode and can ultimately help to expedite the treatment and therapy procedure for CRC patients.

Therefore, in this paper, artificial intelligence-based methods are proposed for automatic classification and automatic localization of abnormal regions in WSI. The proposed models can assist the pathologists and surgeons in faster decision making. The remaining sections of this paper are organized as follows. In Section 2, the dataset and methods used in this paper are discussed, followed by the experiments performed in Section 3. Section 4 is used for discussing the results of both pretrained and customized models along with the localized results. In Section 5, discussions and comparisons of the current works in this field are presented, and finally, the work is concluded in Section 6.

## 2. Materials and Methods

### 2.1. Study Outline

**Figure 1:**
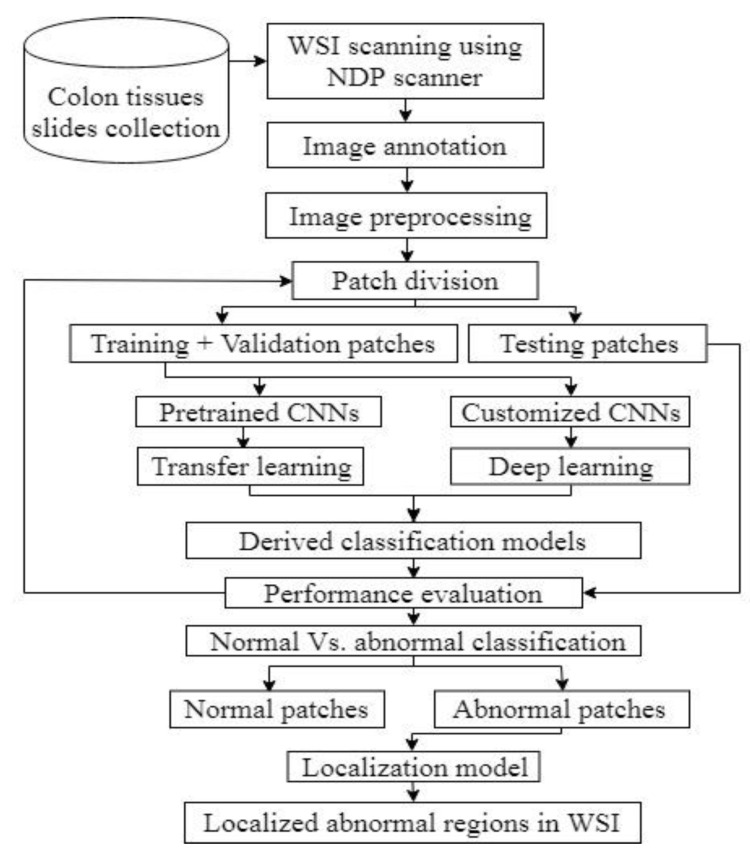
The overall outline of the study.

Figure 1 shows the overall outline of the study, wherein the steps involved from image data collection to scanning and obtaining WSI were briefed. This was followed by preprocessing and the division of data for classification model evaluation. The customized models were evaluated using performance metrics and, finally, abnormal tissues were localized in WSI. All the steps were elucidated in the following subsections.

### 2.2. Data Acquisition

In this work, data were collected from 77 subjects containing 297 WSI for the colon, including both normal and abnormal subjects. All the collected tissue samples were taken between 2009 and 2011 at the Chang Gung Memorial Hospital, Linkou, Taiwan. The samples collected for the study were anonymized and approved by the Institutional Review Board, Chang Gung Memorial Hospital, Linkou, Taiwan, under the license number 201702073B0. The size of the specimen varied among subjects and thus the size of scanned images. These specimens were stained with H&E and scanned using Hamamatsu NanoZoomer at 40× magnification, resulting in a vendor-dependent format named NanoZoomer Digital Pathology (NDP) images. In this study, the largest source size of the specimen obtained was 43.4 mm × 25.6 mm, which led to an NDP image size of 3.18 GB with a resolution of 229 nm/pixel (110,917 dpi) and dimensions of 188,416 × 112,384 pixels.

### 2.3. Image Annotation

After obtaining the digitalized WSI comprising 269 slides for abnormal subjects and 28 slides for normal subjects, we needed to annotate the normal and abnormal tissue regions for using the supervised learning method to train the deep learning models. As a result, in the WSI, the normal and abnormal regions were annotated by a pathologist who has more than 10 years of experience. While analyzing the WSI, we observed that some of the slides were not optimally scanned, which resulted in blurred images when the images were magnified. In addition, the WSI with overlapped and/or folded tissue samples were discarded. As a result, after discarding non-optimal slides, the study consisted of 22 subjects containing 28 normal WSIs and 55 subjects containing 187 abnormal WSIs. The entire image annotations were carried out using the NDP.view2 software, which is a free edition, provided by Hamamatsu Photonics K.K. for viewing the NDP images.

### 2.4. Image Preprocessing

**Figure 2:**
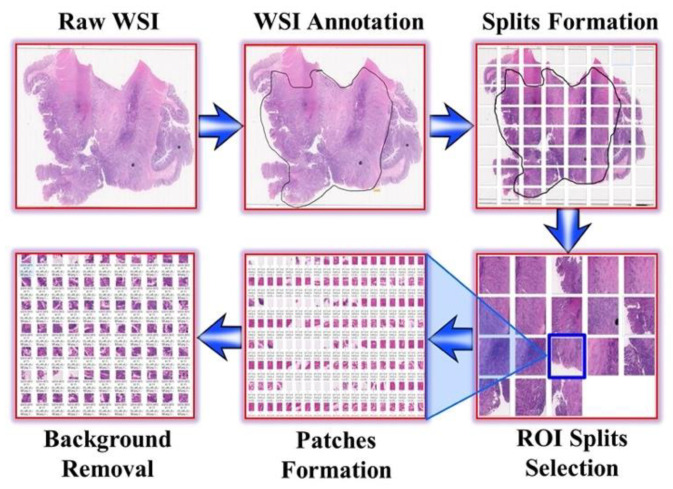
Overall data collection, annotation, and preprocessing method for CRC WSI.

The WSIs obtained for the subjects ranged from 1 to 3 GB, including the images from both normal and abnormal subjects. However, the WSI of such a huge size could not be directly used as input to the deep learning algorithms due to the technical challenge of not being able to fully fit into the computer’s memory. Consequently, NDPITools [22], open-source software distributed under GNU General Public License 3.0, was used for splitting the WSI into smaller splits with an overlap of 25 pixels, resulting in splits of JPEG format. There were 32, 64, or 128 splits formed per WSI, depending on the size of input WSI. When scanned, the WSI also contained white background of the slide, which was not required in the analysis. In addition, some splits consisted of 75–100% overlapping tissues, which were the results of poor fixation during slide preparation. As a result, the splits containing white background, artefactual staining, and tissue wrinkling were discarded manually under the pathologist’s supervision. The remaining splits were used as input to obtain the patch (tile). TileMage Image Splitter 2.11 was used for obtaining patches varying from 200 to 300 pixels by 200 to 300 pixels. After the patch formation, a similar approach was adopted for the removal of patches containing white background and tissue wrinkling, and then, finally, the patches were ready for training the deep learning models. Figure 2 shows the step-by-step procedure adopted from image data collection to preprocessing.

### 2.5. Artificial Intelligence-Based Analysis

In artificial intelligence, among the artificial neural networks, the CNN, which uses convolution operation, weight sharing, and local connectivity principle, is considered best suited for image analysis. Two types of training were considered for the CNNs: the first set for training consisted of pretrained networks and the second set for training consisted of the customized CNNs, which were built taking the different CNN architectures as the base. The CNN is well known for being used in medical image analysis, which learns the important features of an image efficiently, omitting the feature engineering step that is used in a typical machine learning approach. The most important layer in a CNN is the convolutional layer, which applies convolution over the input. Let the input image be denoted by q and the kernel be denoted as r. The output indexes of rows (m) and columns (n) of the resultant feature map will be as given in Equation (1).

(1) $$O\left[m,n\right]=\left(q\times r\right)\left[m,n\right]=\sum_{j}\sum_{k}q\left[j,k\right]r\left[m-j,n-k\right]\;,$$

When applying convolution, the information from pixels located on the outskirts is lost. As a result, padding is used for solving such issues. Depending on whether padding is used or not, the types of padding are valid, meaning that no border is used around the input, and the same, wherein the border is used around the input. When the same padding is applied with filter dimension  r, the padding p must satisfy Equation (2).

(2) $$p=\frac{r-1}{2}\;,$$

After the padding, the dimension of the output feature map ($d_{out}$) or output image can be calculated using Equation (3) as follows:

(3) $$d_{out}=\lfloor \frac{d_{in}+2p-r}{s}+1\rfloor \;,$$

where s is the stride, and $d_{in}$ is the dimension of the input feature map. Let us consider that there are $n_{r}$ filters and the number of channels for the image is $n_{c}$. The dimension of the whole output can be calculated using Equation (4).

(4) $$\left[q,q,n_{c}\right]\times \left[r,r,n_{c}\right]=\left[\lfloor \frac{q+2p-r}{s}+1\rfloor ,\lfloor \frac{q+2p-r}{s}+1\rfloor ,n_{r}\right]\;,$$

When applying the convolution over an input during forward pass, we calculated an intermediate value z using Equation (5) as follows:

(5) $$z=\sum_{i}w_{i}x_{i}+b\;,$$

where $w_{i}$ represents the weight associated with input feature $x_{i}$, and b is the bias. If z, the output produced by one layer, was directly forwarded to the next layer, it led to linear and weak learning. As a result, for introducing non-linearity, an activation function was applied after convolution operation in the convolutional layer itself using Equation (6).

(6) $$z=f\left(\sum_{i}w_{i}x_{i}+b\right)\;,$$

The most preferred activation function, rectified linear unit (*ReLU*), is estimated as given in Equation (7):

(7) ReLU(x)=max(0,x) ,

When performing convolution over an input, not all the outputs produced were important. In addition, the size of the output increased with the increase in filters. Therefore, it was required to downsample the output produced by the convolutional layer. The downsampling was carried out using either max-pooling or average-pooling operation. When using CNN, the convolutional layers with activations followed by pooling layers were applied multiple times as per the requirement. All the layers followed local connectivity. However, for the output of the network to be obtained, all the features must be aggregated, which created the requirement of global connectivity. Therefore, there were fully connected layers attached towards the end of CNN. The fully connected layer works on the multilayer perceptron principle obeying global connectivity among the nodes. Finally, in the output layer, a softmax activation function was used in the last layer to determine the probabilities of each category, using Equation (8):

(8) $$\sigma {\left(z\right)}_{i}=\frac{e^{z_{i}}}{\sum_{j=1}^{K}e^{z_{j}}}\;for\;i=1,2,\ldots ,K.$$

Here, the standard exponential function was applied to each element $z_{i}$ of the input vector z, and normalized by dividing by the sum of all the exponentials.

## 3. Experiments

### 3.1. Data Divisibility

To carry out the image analysis for normal vs. abnormal classification, we obtained 303,012 normal patches and approximately 1,000,000 abnormal patches after all preprocessing steps were completed. In favor of having unbiased performances during the analyses, we performed a five-fold cross-validation study [23] for each pretrained model and customized model, and standard deviation (SD) was also considered. During each round of cross-validation, the dataset was randomly divided into two sets containing training set and testing set in the ratio of 80:20, wherein the training was used for model derivation, and the testing set was used for model evaluation.

### 3.2. Transfer Learning Using Pretrained CNN Architectures

In order to compare the transfer learning with different domain dataset (ImageNet) and to train the models from the scratch using own dataset, we considered different popular pretrained CNNs such as VGG, ResNet, Inception, and IR-v2 for the analysis. In case of pretrained CNN architectures, the weights of ImageNet were used. Therefore, the last layer was only fine-tuned with the considered CRC dataset. While using deep CNNs, we needed to find the most suitable model that could be used for the analysis of histopathology images of the colon. Therefore, different performance metrics [24] such as recall (sensitivity), specificity, precision, accuracy, F-score, and Matthew correlation coefficient (MCC) [25] were considered to evaluate different CNNs. Moreover, the receiver operating characteristic (ROC) curve [26] was plotted showing the AUC, and the average precision (AP) [27] was also calculated.

### 3.3. Deep Learning Using Customized CNN Architectures

By considering the training from the scratch instead of using weights of ImageNet, we built models from the scratch using Keras. The built and compiled model learned only the patterns of histopathology images, and weights were updated in all layers during the learning procedure. The structure of models such as five blocks in VGG16; 1 × 1 and 3 × 3 convolutions in Inception family architectures; skip connections in ResNet50 architecture; and 1 × 1, 3 × 3, and 7 × 7 factorizations with skip connections in IR-v2 were used as the base, and the models were trained from scratch.

### 3.4. Deep Learning Using Variants of Customized Inception-ResNet-v2

**Figure 3:**
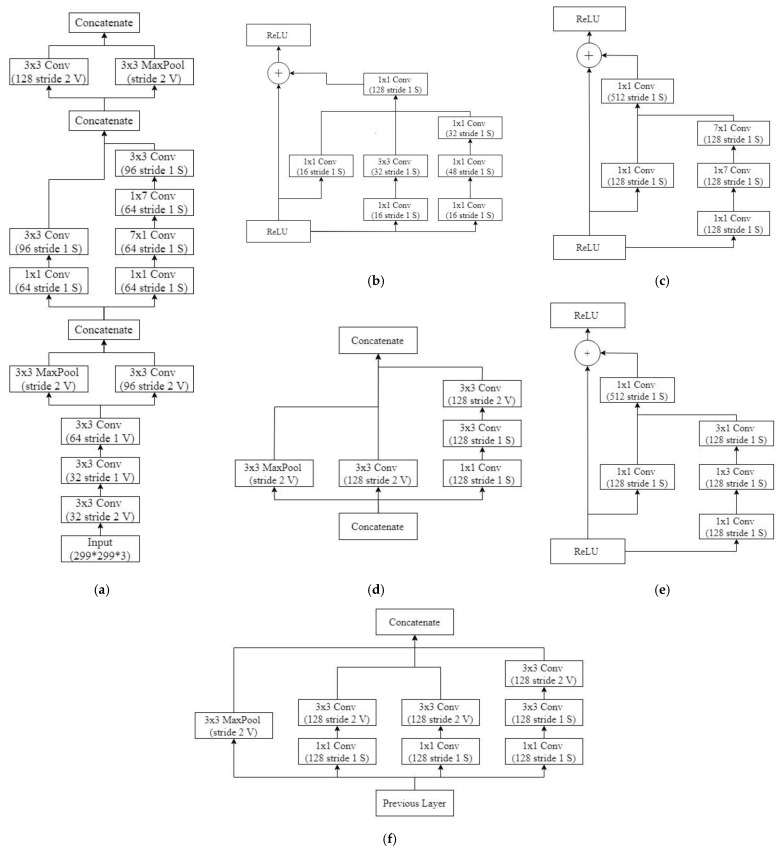
The proposed modular architecture of IR-v2 Type 5: (**a**) Stem; (**b**) Inception-A block; (**c**) Inception-B block; (**d**) 35 × 35 to 17 × 17 reduction module A; (**e**) Inception-C block; and (**f**) 17 × 17 to 8 × 8 reduction module B.

On the basis of the modifications made in the IR-v2 model, we discuss five types of customized IR-v2 models in this study. In Type 1 of IR-v2, the default configuration of the network was used. However, the originally used numbers of linear filters in Inception-A (384), Inception-B (1154), and Inception-C (2048) blocks were reduced to 128 in every block in the case of Type 2 of IR-v2. Moreover, the numbers of filters were also reduced to 128 in every convolutional layer defined in the reduction modules. In addition to the configuration of the network in Type 2, the number of modules for Inception-B was reduced from 10 to 5 in the network used in Type 3. However, in the network used in Type 4, the activation function was changed from softmax to sigmoid in the output layer, keeping the configurations same as the network used in Type 3. On the contrary, the originally used numbers of linear filters in Inception-A (384), Inception-B (1154), and Inception-C (2048) blocks were reduced to 128, 512, and 512, respectively in the case of Type 5. The detailed structure of the best performing model IR-v2 Type 5 is shown in Figure 3, wherein the considered numbers of filters, stride, and pooling are mentioned for the considered structures of Stem in Figure 3a, Inception-A block in Figure 3b, Inception-B block in Figure 3c, 35 × 35 to 17 × 17 reduction module A in Figure 3d, Inception-C block in Figure 3e, and finally 17 × 17 to 8 × 8 reduction module B in Figure 3f. In addition to changing the numbers of filters and layers, modifications were made in the considered number of modules, such as the number of modules in Inception-B being reduced from 10 to 7.

### 3.5. Localization

**Figure 4:**
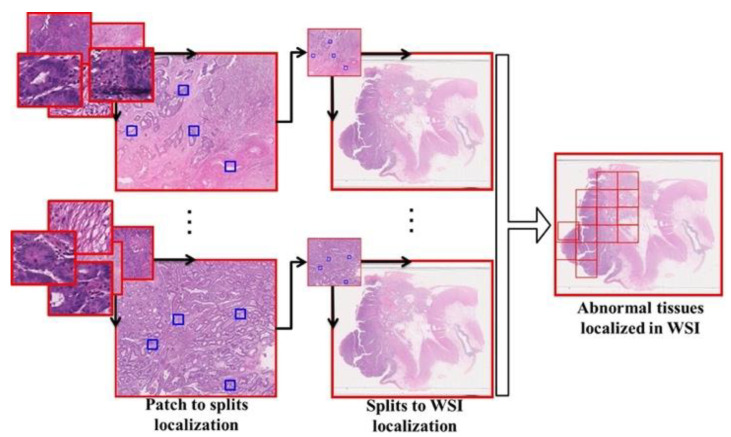
Overall localization in WSI.

When considering the automatization of WSI analysis, we found that the patch level classification was not enough to assist the pathologists. To reduce the burden of pathologists, the abnormal tissues must be exactly localized in the WSI. Therefore, the template matching algorithm was used to exactly localize the abnormal split in the WSI. Template matching is a popular digital image processing technique used for matching a small part of an image referred to as a template (T) to the source image (I). As shown in Figure 4, in this work, the template matching consisted of two phases, wherein in the first phase, only the abnormal patches formed from the splits were localized in the respective splits of varying dimensions. In the second phase, this was followed by the localization of the splits in the WSI, showing the abnormal regions in the WSI of colon tissue. The localization method used in this work is the normalized correlation method [28], calculated using Equation (9).

(9) $$R\left(x,y\right)=\frac{\sum_{\left(\overset{´}{x},\overset{´}{y}\right)}\left(T\left(\overset{´}{x},\overset{´}{y}\right)\cdot I\left(x+\overset{´}{x},y+\overset{´}{y}\right)\right)}{\sqrt{\sum_{\left(\overset{´}{x},\overset{´}{y}\right)}(T{\left(\overset{´}{x},\overset{´}{y}\right)}^{2}\cdot \sum_{\left(\overset{´}{x},\overset{´}{y}\right)}I{\left(x+\overset{´}{x},y+\overset{´}{y}\right)}^{2})}}\;\;,$$

**Figure 5:**
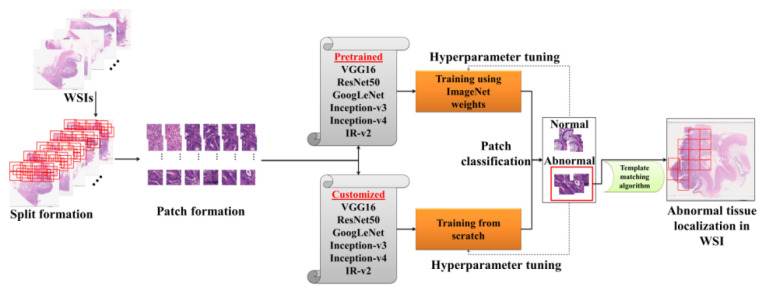
Illustration of the proposed work for classification and localization of abnormal tissues in WSI.

Figure 5 illustrates the proposed work—the patches generated after preprocessing were used for differentiating the abnormal tissues, wherein both pretrained models and customized models were used individually. Finally, the abnormal tissues differentiated by the model were ultimately localized in the WSI using the template matching algorithm.

### 3.6. Implementation Environment

All the classification and localization models were implemented using Tensorflow [29] 1.14, computed on a Linux OS GPU with the specification TITAN RTX 24 GB×4, Intel^®^Xeon^®^Scalable Processors, 3 UPI up to 10.4 GT/s, with 256 GB memory, Nvidia-smi 430.40 in Ubuntu 18.04.3 platform. The other libraries used were Keras 2.1.6, python 3.6.9, numpy 1.18.4, matplotlib 3.2.1, OpenCV 4.1, pillow 7.1.2, and scikit-learn 0.21.3.

## 4. Results

### 4.1. Transfer Learning Using Pretrained CNN Architectures

**Figure 6:**
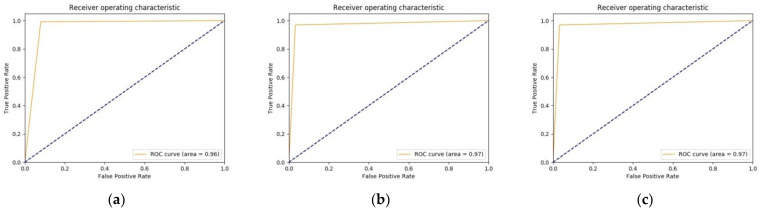

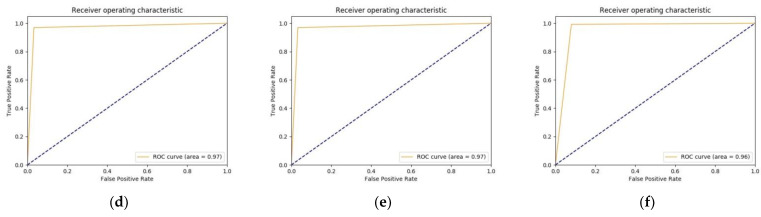
ROC curves for different considered pretrained models: (**a**) VGG16; (**b**) ResNet50; (**c**) GoogLeNet; (**d**) Inception-v3; (**e**) Inception-v4; and (**f**) IR-v2.

When using the weights of ImageNet and fine-tuning the last layer in the case of considered pretrained CNN architectures, we trained the models with learning rate (0.0001), batch size (256), and the number of iterations (20,000). After every 400 iterations, validation was performed. The training times for the models were similar, approximately 2 h ± 15 min for 400 iterations, resulting in approximately 20 s per iteration. Similarly, the validation time was approximately 3 min. The results for different metrics with SD, obtained after performing a fivefold cross-validation study, are shown in Table 1. On the basis of the values obtained by CNN architectures, we observed that VGG16 had the highest sensitivity (0.99 ± 0.012). However, the specificity was lower, which resulted in a lower AUC (0.96), as justified by Figure 6a. Similar performance in terms of AUC can be seen in the case of the model IR-v2, as shown in Figure 6f. On the other hand, ResNet50 and Inception family performed with AUC (0.97), as shown in Figure 6b–e. In general, as per Table 1 and Figure 6, all the models showed similar performances. As a result, further studies were conducted for verifying if the performances of models could be improved when the models were trained from scratch with the same domain dataset.

### 4.2. Deep Learning Using Customized CNN Architectures

When considering the creation, compilation, and training of models from scratch, we trained all the customized models with initial learning rate (0.0008), batch size (128), number of epochs (50), and the optimizer used was Adam. Moreover, the input size was 224 × 224 for VGG16, ResNet50, and GoogLeNet, and 299 × 299 for Inception-v3, Inception-v4, and IR-v2. All the customized models took approximately 5 days when trained from scratch. The performances of the different customized models are presented in Table 2. On the basis of the values of performance metrics such as F-score, VGG16 achieved 0.89 ± 0.002, which was reduced by 0.06 when compared to F-score achieved in Table 1 (0.95 ± 0.00). Similar observations were made in the case of all other models (except IR-v2), wherein the F-score was reduced by 0.17 (ResNet50), 0.10 (GoogLeNet), 0.03 (Inception-v3), and 0.12 (Inception-v4) when the models were trained from scratch.

**Figure 7:**
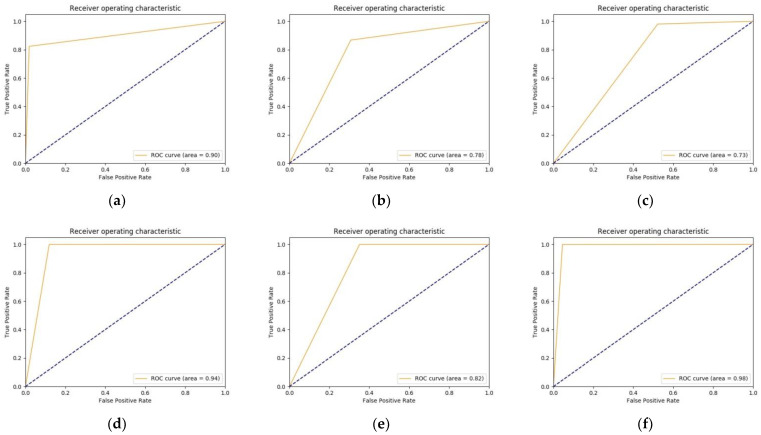
ROC curves for different considered customized models: (**a**) VGG16; (**b**) ResNet50; (**c**) GoogLeNet; (**d**) Inception-v3; (**e**) Inception-v4; and (**f**) IR-v2.

The declines in performances were further justified by ROC curves for the different customized models, as shown in Figure 7. On the basis of the observations, we found that the performance of VGG16 reduced in the customized model, which is reflected in AUC achieved in Figure 7a, and AP also reduced from 0.91 ± 0.003 (Table 1) to 0.89 ± 0.013 (Table 2). Similar observations were made in the case of another model where AUC and AP were reduced to 0.78 (Figure 7b) and 0.71 ± 0.017 (Table 2), respectively, for ResNet50. Moreover, AUC and AP were respectively reduced to 0.73 (Figure 7c) and 0.74 ± 0.013 (Table 2) for GoogLeNet, 0.94 (Figure 7d) and 0.89 ± 0.019 (Table 2) for Inception-v3, and 0.82 (Figure 7e) and 0.74 ± 0.018 (Table 2) for Inception-v4.

### 4.3. Deep LEARNING Using Variants of Customized Inception-ResNet-v2

Among all the customized CNNs, as observed from Table 2 and Figure 7, the customized IR-v2 performed better than all other models in terms of accuracy, sensitivity, F-score, etc. Therefore, for further analysis, IR-v2 was considered as the base model, and several modifications were made such as changing the input image size, the number of hidden layers, and the numbers of filters in hidden layers, as already elucidated in Section 3.4. The training parameters for the models are given in Table 3.

Among all the variants of customized IR-v2, IR-v2 Type 5 performed better in comparison to other types, as observed from the values of different performance metrics represented in Table 4. The model achieved an F-score of 0.99 ± 0.005 in a fivefold cross-validation study [23], with minimum SD, demonstrating no overfitting issue in the model derivation.

**Figure 8:**
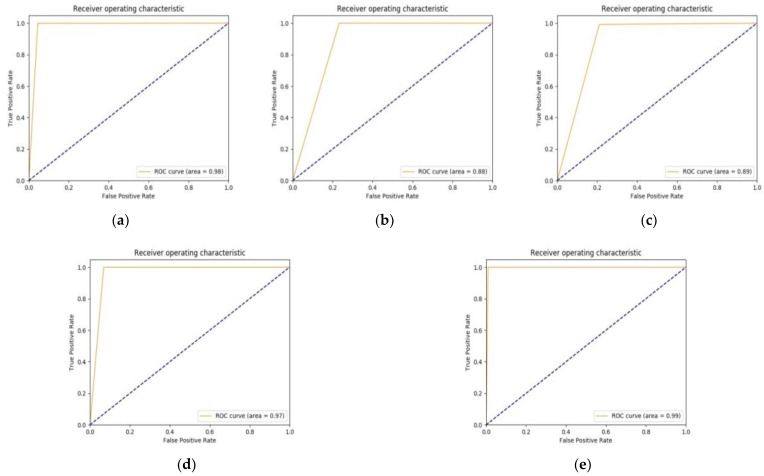
ROC curves for types of different customized IR-v2: (**a**) IR-v2 Type 1; (**b**) IR-v2 Type 2; (**c**) IR-v2 Type 3; (**d**) IR-v2 Type 4; and (**e**) IR-v2 Type 5.

The better performance of IR-v2 Type 5 can be also justified by the ROC curves plotted in Figure 8e, which shows an AUC of 0.99, which is followed by the next best performing model, the Type 1 of IR-v2 with AUC 0.98, as observed in Figure 8a. The remaining models achieved AUC 0.88 (Figure 8b), 0.89 (Figure 8c), and 0.97 (Figure 8d) for Type 2, Type 3, and Type 4, respectively.

### 4.4. Classification and Localization Results

#### 4.4.1. Classification Results

**Figure 9:**
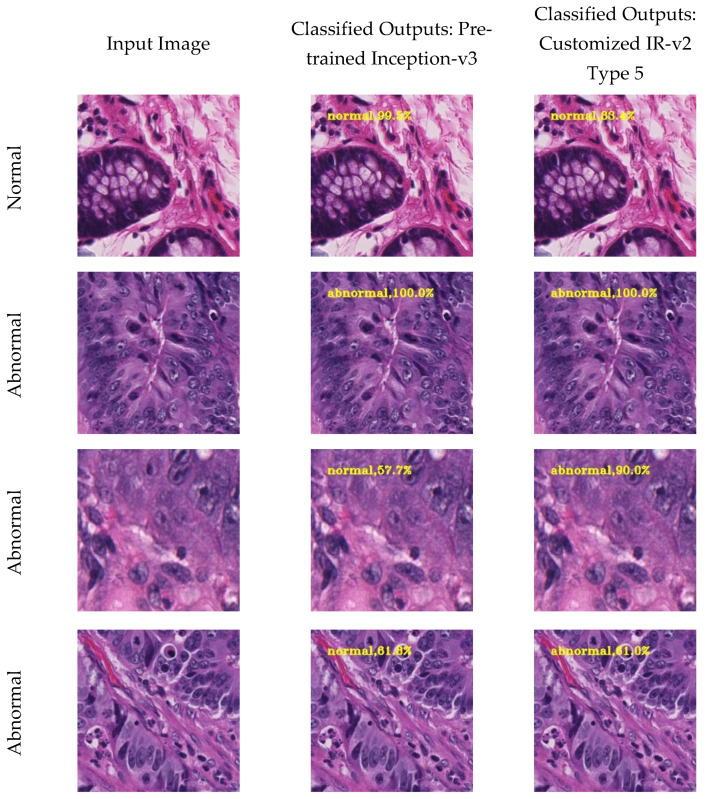
Classification results produced by the models with own dataset: column 1: input image; column 2: classified outputs from pretrained Inception-v3; and column 3: classified outputs from IR-v2 Type 5.

In order to justify that the performance of IR-v2 Type 5 is better than the Inception-v3, which is the best performing model among the pretrained CNNs, we trained the former from scratch, and the latter used the weights of ImageNet for transfer learning. Some of the outputs produced by both the models are displayed in Figure 9. The first column of Figure 9 represents the input image for the models, wherein the first image in column 1 is the normal patch and the remaining three images are abnormal patches. The outputs produced by the pretrained Inception-v3 and customized IR-v2 Type 5 are shown in column 2 and column 3, respectively. Here, Inception-v3 misclassified two abnormal patches, row 3 and row 4, as normal. However, IR-v2 Type 5 could correctly classify both the patches as abnormal, as shown in row 3 and row 4.

#### 4.4.2. Localization Results

**Figure 10:**
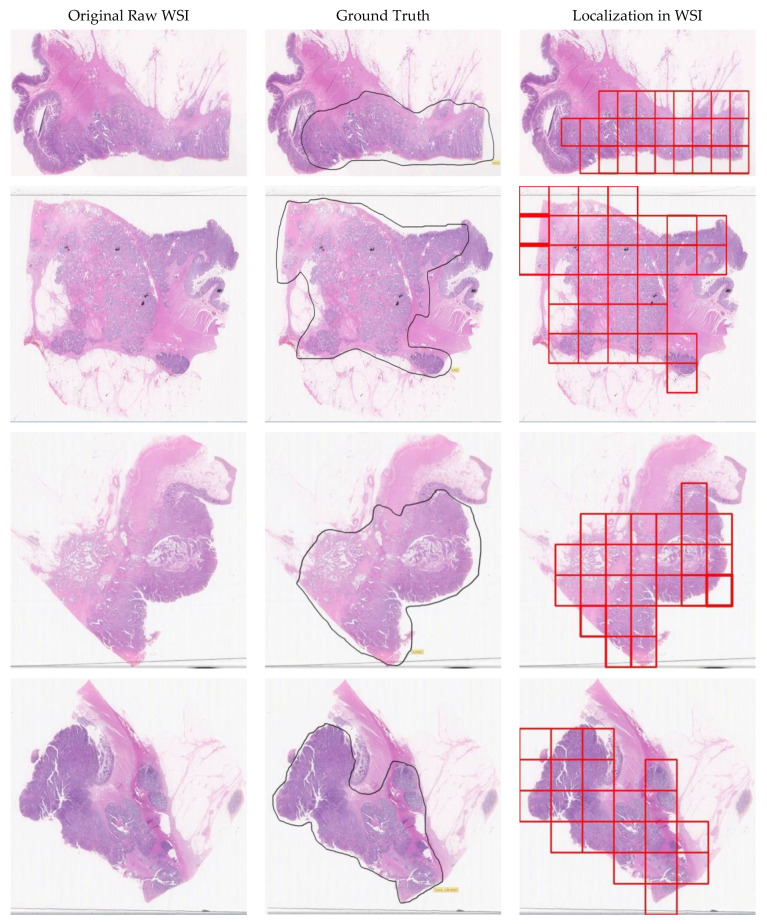
Comparison of ground truths vs. the outputs produced by IR-v2 Type 5: column 1: original WSIs; column 2: corresponding ground truths; column 3: corresponding output for abnormal tissues localized in WSIs.

The localization results of abnormal tissues in WSI are shown in Figure 10, where column 1 shows the digitally scanned original WSIs of different subjects. Column 2 shows the annotated ground truths. Finally, column 3 shows the localized abnormal tissues output obtained after classification from the IR-v2 Type 5. It can be observed that the IR-v2 Type 5 can accurately localize the abnormal tissues in the WSI, thereby minimizing the burden of pathologists during tissue examination.

## 5. Discussion

### 5.1. Comparison with Previous Works in the Same Domain

When considering the analysis for different cancers and diseases such as kidney disease [30], brain tumor, prostate cancer, and colon cancer [31], researchers have carried out several studies for automatic and semi-automatic analysis using AI [32]. In particular, considering the histology images analysis, there have been several research works conducted for not only colorectal cancer, but also different types of cancers [33], such as breast cancer, skin cancer [34], and renal cancer [35]. Considering the manual method of feature extraction in the histology image analysis of CRC, the authors in [36] performed the ML-based differentiation of colorectal tissue with and without adenocarcinoma, using quasi-supervised learning. Statistical and texture features were extracted from 230 images of colorectal tissues, wherein the dimension of considered images was 4080 × 3720 pixels. The accuracy achieved by the model for the binary classification was 95%. In [37], the authors considered 20 WSI of CRC tissue for generating patch size of 150 × 150 pixels for training, and patch size of 5000 × 5000 pixels for testing. The patches were used to obtain the different statistical and texture features and train the ML models for performing eight class classifications. The authors used one nearest neighbor; support vector machine (SVM), and decision tree as the ML classifiers, and the best accuracy achieved was 87.4%. However, when considering ML analysis, the features are handcrafted. Manual feature extraction is a tedious job, and some unknown important features might be overlooked.

Therefore, towards the end of 2016, many works were published that focused on the use of DL for image analysis [38]. One of the works [39] used CNN to extract the features automatically, and the extracted features were used for the classification of breast and colon cancer into benign and malignant tumors. The CNN model consisted of five layers of architecture similar to LeNet and achieved 99.74% accuracy for binary classification. With a focus on colorectal histology image analysis, a binary classification model was proposed using VGG as the base model. The work used 28 normal and 29 tumor images, and cropped into 6806 normal and 3474 tumor images, achieving sensitivity, specificity, and accuracy of 95.1%, 92.76%, and 93.48%, respectively. The derived best-modified model was able to correctly classify 294 out of 309 normal images, as well as 667 out of 719 tumor images [40]. Similarly, the work in [41] focused on DL analysis for omitting the feature engineering and performing the classification of colorectal cancer into benign or malignant on the basis of tumor differentiation and classifications of tumor in the brain and colorectal tissues into normal and abnormal considering 717 patches and using AlexNet architecture, achieving 97.5% accuracy for classification. However, they used an SVM classifier instead of using softmax for the classification.

A different contribution was made in [42], wherein the authors attempted to predict the 5-year disease-free survival (DFS) in the case of patients with CRC. The work used VGG16 for feature extraction and long short-term memory for predicting the 5 years survival probability. However, the work achieved an AUC of only 0.69 when performing the DFS prediction directly from the image. Recently, the authors in [43] trained CNNs and recurrent neural networks on WSI of stomach and colon for performing multiclass classification considering three categories, namely, adenoma, adenocarcinoma, and non-neoplastic. They achieved AUC up to 0.99 and 0.97 for the gastric adenoma and adenocarcinoma, respectively. On the other hand, for colonic adenoma and adenocarcinoma, AUC 0.99 and AUC 0.96 were achieved, respectively. Considering 170,099 patches obtained from around 14,680 WSIs of more than 9631 subjects, the first-ever huge generalizable AI system was developed in [44]. The system used a novel patch aggregation strategy for the CRC diagnosis using weakly labeled WSI, wherein the Inception-v3 was used as the architecture with weights initialized from the transfer learning. The AI system generated output in the form of a heatmap highlighting cancers tissue/cells in WSI. Considering the various works proposed for the histopathological image analysis of colon cancer, we present a summary table (Table 5) showing the performances of different works in terms of one or more metrics such as accuracy, sensitivity, and AUC.

### 5.2. Comparison of Different CNN Architectures Taking Public Dataset

As presented in Table 5, a recent study [12] used TL with EfficientNet for the classification of breast cancer images and achieved an accuracy of 98.33%. Furthermore, another study [44] used TL with Inception-v3 and achieved AUC 0.988 for CRC classification. The achievements of both works were comparable to the performance of the IR-v2 Type 5 model. As a result, a public dataset [45] was used to compare the performances of pretrained Inception-v3 and EfficientNet with IR-v2 Type 5 to verify the robustness of the latter. The validation dataset consisted of nine classes. However, only 741 images of normal and 1233 images of tumor (abnormal) were used. The performance metrics of different models are shown in Table 6, wherein IR-v2 Type 5 is shown to have performed better, with an accuracy of 90% and F-score of 91%.

**Figure 11:**
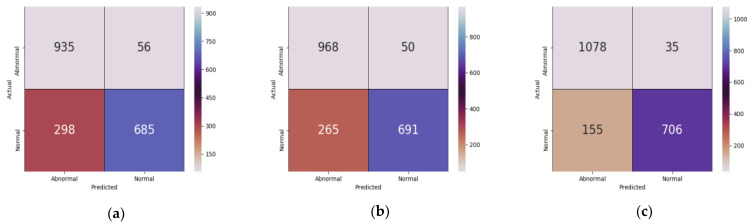
Confusion matrices considering public dataset [45]: (**a**) EfficientNet, (**b**) Inception-v3, and (**c**) IR-v2 Type 5.

**Figure 12:**
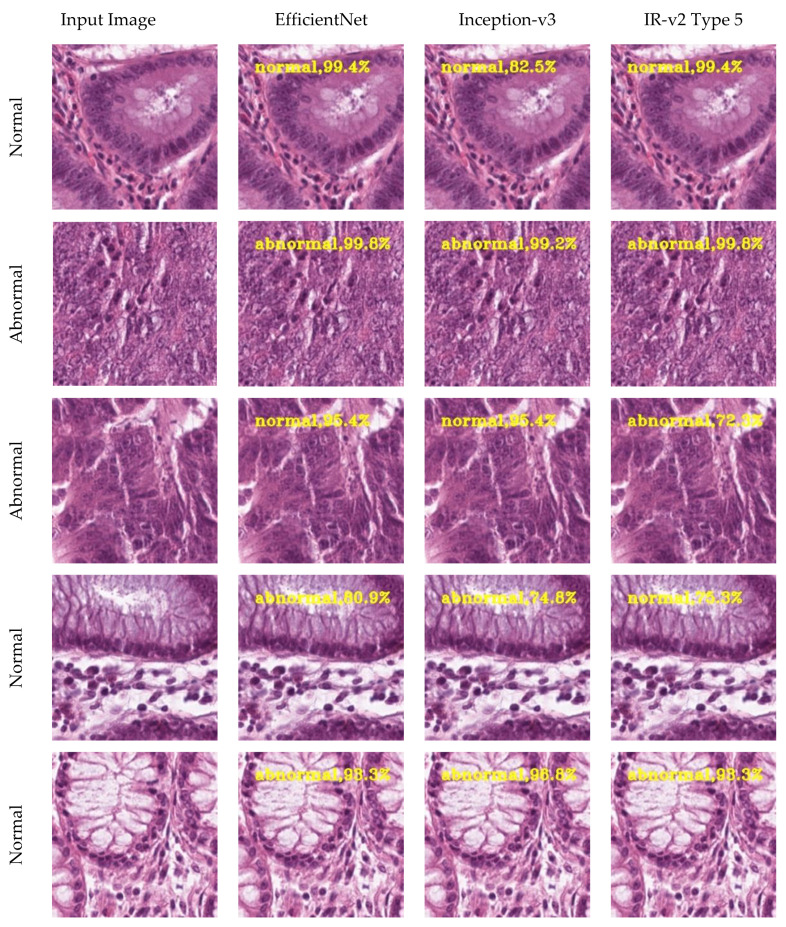
Comparison of outputs generated by the models: column 1: input image; column 2: classified outputs produced by EfficientNet; column 3: classified outputs produced by Inception-v3; and column 4: classified outputs produced by IR-v2 Type 5.

Furthermore, the confusion matrices for the models were presented in Figure 11, which shows that IR-v2 Type 5 could identify the tumor images more correctly as compared to other models, which is illustrated in Figure 12, wherein outputs produced by different models are presented. The first column represents the input image, while the second, third, and fourth columns contain the classified outputs produced by EfficientNet, Inception-v3, and IR-v2 Type 5, respectively. On the basis of Figure 12, all three models were correctly classified the outputs in the case of input images presented in the first and second rows. However, both EfficientNet and Inception-v3 failed to correctly classify the input images as given in the third and fourth row, although IR-v2 Type 5 could also correctly classify these two images. However, in the case of the input image given in row 5, all models were incorrectly classified the normal images as abnormal. In terms of the results in Table 6, confusion matrices depicted in Figure 11, and outputs from Figure 12 showed that IR-v2 Type 5 also performed better when the public dataset was used.

### 5.3. Processing Time and Visualization of Intermediate Outputs

By considering a model for the medical image analysis, we needed to ascertain that not only the model should produce better output, but also it should be faster in producing the output. Therefore, Table 7 shows the time details related to the model training for 50 epochs, single epoch processing time, and individual image testing time considering our own dataset. The derived IR-v2 Type 5 model could classify each image within 0.58 s, which is significantly faster. This could be further improved using GPU with more powerful configuration than the currently used GPU.

**Figure 13:**
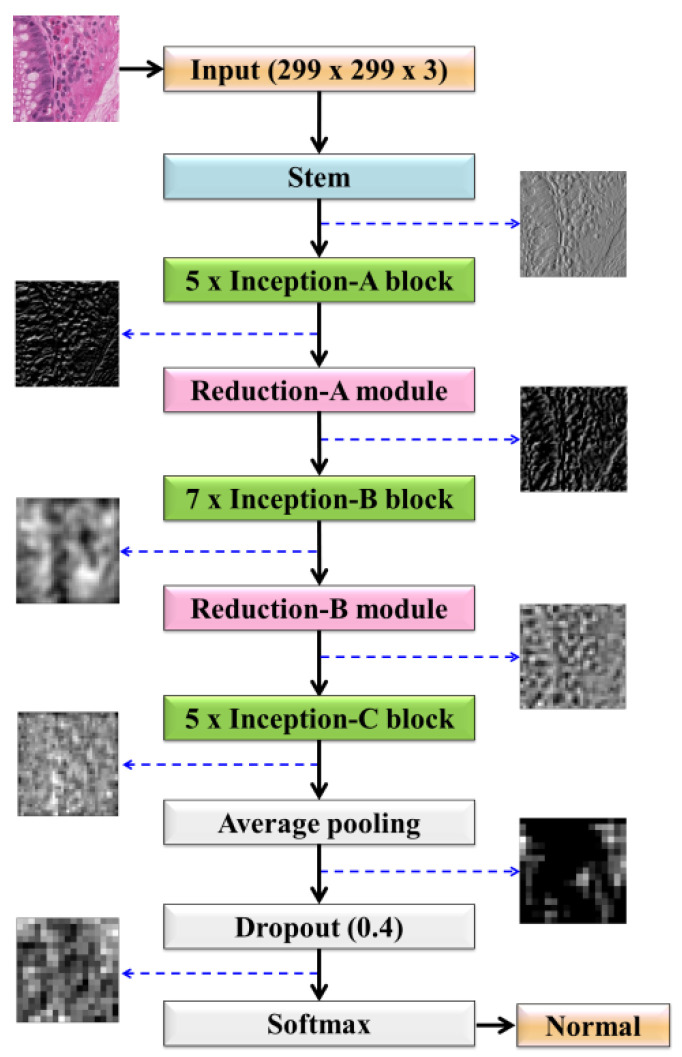
Visualization of intermediate outputs produced by different blocks of IR-v2 Type 5.

In addition, Figure 13 shows the outputs of the learned features in different blocks of the model IR-v2 Type 5, where initially the model learned the low-level features such as edges and colors. Gradually, the model captured the complex features, which are less interpretable and became more abstract as the learning moved towards the output layers.

Considering different works proposed as above, this paper proposed a comparative study considering different pretrained CNN and customized CNN. Moreover, this work mainly focused on developing a model using customized CNN architecture that performed well on the digital histopathology WSI data, especially CRC. Consequently, the IR-v2 Type 5 model developed through this study could be deployed in different hospitals for automatic classification and localization of abnormal tissues in WSI, which can assist the pathologists in making the faster and accurate decision, which could ultimately expedite the treatment and therapy procedure of CRC patients. In addition, the models could be used for other cancer decisions by fine-tuning them with samples of other types of cancer images.

## 6. Conclusions

In this paper, the IR-v2 Type 5 model was designed for distinguishing the normal and abnormal patches obtained from the WSI of the colon cancer patients. Moreover, the abnormal regions were localized in the WSI. It was observed that in the case of the pretrained CNN architecture, the Inception-v3 performed better in comparison to other models with an F-score of 0.97. However, when the different models were customized in terms of numbers of filters, size of the input image, and/or the number of hidden layers, and trained from scratch, it was concluded that the IR-v2 Type 5 model achieved an F-score and AUC of 0.99. With automatic classification and localization of the abnormal tissues in WSI, the workload of the pathologists would be reduced and faster decisions for the treatment procedures could be made. However, there are some limitations in our study such as the population considered in this study representing only a specific region. In addition, the slides used for collecting the WSI belonged to a single hospital; as a result, we plan to consider a multi-institutional study, wherein data would be collected from different hospitals and analyzed to improve the robustness of the derived IR-v2 Type 5 model. Furthermore, the future work includes the scope where the designed model may not only detect and localize the abnormal region in the WSI but also can determine the primary tumor growth for the determination of the stages of CRC.

## Figures and Tables

**Table 1:** Pretrained models with performance metrics along with SD.

Metrics	VGG16	ResNet50	GoogLeNet	Inception-v3	Inception-v4	IR-v2
Sensitivity	0.99 ± 0.012	0.96 ± 0.008	0.95 ± 0.011	0.97 ± 0.011	0.97 ± 0.019	0.94 ± 0.032
Specificity	0.92 ± 0.013	0.96 ± 0.019	0.97 ± 0.011	0.96 ± 0.014	0.95 ± 0.003	0.96 ± 0.023
Precision	0.97 ± 0.013	0.96 ± 0.019	0.97 ± 0.011	0.96 ± 0.008	0.95 ± 0.003	0.97 ± 0.025
Accuracy	0.95 ± 0.00	0.96 ± 0.004	0.96 ± 0.00	0.96 ± 0.010	0.96 ± 0.008	0.95 ± 0.005
F-score	0.95 ± 0.00	0.96 ± 0.004	0.96 ± 0.00	0.97 ± 0.005	0.96 ± 0.008	0.95 ± 0.005
MCC	0.94 ± 0.014	0.93 ± 0.005	0.91 ± 0.015	0.93 ± 0.005	0.93 ± 0.018	0.89 ± 0.003
AP	0.91 ± 0.003	0.95 ± 0.013	0.96 ± 0.003	0.95 ± 0.009	0.95 ± 0.018	0.96 ± 0.01

**Table 2:** Customized models with performance metrics along with SD.

Metrics	VGG16	ResNet50	GoogLeNet	Inception-v3	Inception-v4	IR-v2
Sensitivity	0.82 ± 0.021	0.86 ± 0.018	0.88 ± 0.031	0.99 ± 0.014	0.99 ± 0.009	0.99 ± 0.014
Specificity	0.98 ± 0.031	0.69 ± 0.023	0.67 ± 0.031	0.88 ± 0.014	0.65 ± 0.013	0.95 ± 0.023
Precision	0.97 ± 0.031	0.83 ± 0.023	0.85 ± 0.031	0.89 ± 0.028	0.88 ± 0.053	0.95 ± 0.031
Accuracy	0.90 ± 0.001	0.78 ± 0.014	0.82 ± 0.018	0.94 ± 0.011	0.82 ± 0.048	0.97 ± 0.014
F-score	0.89 ± 0.002	0.79 ± 0.009	0.86 ± 0.192	0.94 ± 0.015	0.84 ± 0.048	0.97 ± 0.024
MCC	0.74 ± 0.019	0.61 ± 0.005	0.64 ± 0.025	0.93 ± 0.015	0.8 ± 0.018	0.97 ± 0.021
AP	0.89 ± 0.013	0.71 ± 0.017	0.74 ± 0.013	0.89 ± 0.019	0.74 ± 0.018	0.96 ± 0.014

**Table 3:** Training parameters for variants of customized IR-v2.

Parameter	Value
Batch size	128
# of epochs	50
Optimizer	Adam
Momentum	0.9
Learning rate	0.0008
Dropout	0.4

**Table 4:** Different types of IR-v2 with performance metrics along with SD.

Metrics	IR-v2 Type 1	IR-v2 Type 2	IR-v2 Type 3	IR-v2 Type 4	IR-v2 Type 5
Sensitivity	0.99 ± 0.014	1.00 ± 0.054	0.99 ± 0.064	0.97 ± 0.012	0.99 ± 0.002
Specificity	0.95 ± 0.023	0.76 ± 0.038	0.78 ± 0.064	0.97 ± 0.011	0.99 ± 0.004
Precision	0.95 ± 0.031	0.88 ± 0.044	0.90 ± 0.054	0.97 ± 0.012	0.99 ± 0.003
Accuracy	0.97 ± 0.014	0.88 ± 0.054	0.89 ± 0.034	0.97 ± 0.010	0.99 ± 0.005
F-score	0.97 ± 0.024	0.89 ± 0.044	0.90 ± 0.064	0.97 ± 0.010	0.99 ± 0.005
MCC	0.97 ± 0.021	0.87 ± 0.044	0.87 ± 0.032	0.94 ± 0.014	0.99 ± 0.003
AP	0.96 ± 0.014	0.81 ± 0.054	0.82 ± 0.052	0.96 ± 0.014	0.99 ± 0.001

**Table 5:** Comparison of previous methods with the proposed IR-v2 Type 5 considering colon cancer histopathological WSI.

Reference	Method	Results
[32]	ML-based feature extraction	Accuracy: 98.07%
[36]	Quasi supervised learning	Accuracy: 95%
[37]	Multi-class texture analysis	Accuracy: 84%
[39]	DCNN	Accuracy: 100% F1-score: 100% MCC: 100%
[40]	VGG-variant	Accuracy: 93.48% Sensitivity: 95.1% Specificity: 92.76%
[41]	AlexNet	Accuracy: 97.5%
[42]	VGG16 + LSTM	AUC: 0.69
[43]	CNN + RNN	AUC: 0.99
[44]	Inception-v3	AUC: 0.988
Proposed model	IR-v2 Type 5	AUC: 0.99

**Table 6:** Comparison of recent methods with our proposed method taken from a public dataset [45].

Metrics	EfficientNet [12]	Inception-v3 [44]	IR-v2 Type 5
Sensitivity	75%	78%	87%
Specificity	92%	93%	95%
Precision	94%	95%	96%
Accuracy	82%	84%	90%
F-score	84%	86%	91%

**Table 7:** Processing time details for the IR-v2 Type 5 model.

Type of Time	Durations
Training time	5 days, 7 h, 12 min, 38 s
Single epoch execution time	2 h, 17 min, 21 s
Single image testing time	0.58 s

## Data Availability

The data are not publicly available due to privacy issues of the patients.
